# Chinese Versus United States Workplace Ageism as GATE-ism: Generation, Age, Tenure, Experience

**DOI:** 10.3389/fpsyg.2022.817160

**Published:** 2022-02-17

**Authors:** Michael S. North

**Affiliations:** Department of Management and Organizations, Stern School of Business, New York University, New York, NY, United States

**Keywords:** ageism, cross-cultural, workplace, generation, age, tenure, experience

## Abstract

Ageism is a pan-cultural problem, and correspondingly, increased research attention worldwide has focused on how a person’s age drives prejudice against them. Nevertheless, recent work argues that chronological age alone is a limited predictor of prejudice—particularly in the workplace, where age conflates intertwined elements (e.g., life stage and work experience), and across cultures, in which the nature of ageism can substantially differ. A recent organizational behavior (GATE) framework advocates for extending beyond numerical age alone, focusing instead on prejudice arising from workers’ perceived *Generation* (birth cohort), *Age* (life stage), *Tenure* (time with organization), and *Experience* (accumulated skillset over time). In addition to clarifying the multifaceted nature of workplace ageism, GATE helps uncover potential cultural ageism differences. Using the United States and China as focal Western and Eastern prototypes, the current paper compares Eastern and Western cultures through a GATE Lens. Eastern and Western cultures adopt different perceptions of generations (e.g., United States “Boomers,” versus Chinese “Cultural Revolution” generation), elder life stages (United States warm-but-incompetent older adults, versus Eastern pragmatic elder resource concerns), organizational tenure expectations (Western job-hopping, versus Eastern filial-piety-based loyalty), and desired experience levels (shaped different by higher Eastern frequency of mandatory retirement practices and family business ventures). Moreover, existing research offers clues for how workplace GATE-ism likely differs between cultures, but more research is needed. Future research should adopt a nuanced GATE conception of “age”-ism, toward enhanced ageism understanding and the ability to utilize a globally aging workforce.

## Introduction

From both a research and real-world standpoint, the issue of ageism recently has begun to “come of age.” After a number of early millenium papers noted ageism’s lack of attention compared to sexism and racism (Nelson, 2005; North and Fiske, 2012), the past decade-plus has witnessed a spike in topical interest, relevance, and research (Zhang et al., 2016; Ayalon and Tesch-Romer(eds), 2018). Likewise, organizational scholars increasingly note that an aging and multigenerational workforce warrants a corresponding rise in research attention (Joshi et al., 2010, 2011; Hedge and Borman, 2012; Kulik et al., 2014; North, 2019). From an organizational standpoint, too, a steady increase in age discrimination charges—at least in the United States (North and Fiske, 2015a)—has rendered the issue difficult to ignore.

The spike in interest has gone global, as well, as various studies have begun to explore ageism’s impact cross-culturally. As one piece of evidence, a current google scholar search (conducted on December 17, 2021) using the term “cross-cultural ageism” yields 8,300 total results through the year 2000. Since then, that number has more than doubled, with 17,700 results comprising articles published from the year 2001 through the present. Integrating these findings, one recent meta-analysis comparing Eastern and Western attitudes toward older adults (North and Fiske, 2015b) finds Easterners to harbor more negative perceptions of older adults than Westerners, predicted by recent dramatic rises in population aging, and (perhaps counterintuitively) heightened levels of collectivism. Notably, only two of the analyzed studies in this comprehensive analysis were published before 1996, suggesting that cross-cultural ageism remains a fairly nascent subtopic. Further signaling that scholars have more to unpack within this domain, other work has found that older adults are indeed viewed more positively in the East than in the West, at least as gauged by the Implicit Association test and a one-item explicit measure of age group preference (1 = I strongly prefer Old People to Young People; 5 = I strong prefer Young People to Old People; Ackerman and Chopik, 2021). Thus, cross-cultural ageism is a sub-literature still in need of greater nuance, particularly in terms of unearthing the psychological underpinnings of ageism.

Further complicating the picture is the question what is it about “age” that fosters such prejudice. The most notable research in this realm focuses on age in the workplace, whereby researchers have questioned the value of chronological age as a predictor of key outcomes; for instance, in predicting worker performance and values, subjective age (i.e., how old one *feels* versus how old one is) might predict outcomes above and beyond chronological age alone (Kunze et al., 2015; Nagy et al., 2019). Likewise, from a prejudice standpoint, age conflates intertwined elements (generation, age/lifestage, tenure, and experience; GATE), each of which offers sometimes-countervailing ageism predictions (i.e., prejudice against “older workers” may be exacerbated toward “Boomers” but attenuated toward “experienced workers”). Thus, disentangling GATE elements potentially offers a more nuanced portrait of how prejudice differentially targets different workers of the same chronological age (North and Shakeri, 2019).

To these ends, and toward elucidating the nature of cross-cultural workplace ageism, the current paper proposes research trajectories, comparing China and the United States as Eastern and Western prototypes. In so doing, this paper builds upon a recent organizational behavior GATE framework, emphasizing key distinctions between workers’ perceived *Generation* (based on birth cohort), *Age* (based on life stage), *Tenure* (based on time with current organization), and *Experience* (based on skill accumulation over time; North, 2019). Originally proposed as a framework toward better understanding older workers, GATE potentially offers a better understanding of what underlies “age” prejudice toward different types of older workers (e.g., different forms/levels of prejudice likely target the “older career switcher” embarking on a new career path, versus the “elder incumbent” who has remained with their current organization for decades). Much as GATE clarifies why chronological age alone cannot account for differences in older worker outcomes, the current paper clarifies why a one-size-fits-all lens of workplace ageism should not be applied in blanket fashion across cultures. Through a GATE lens, the current paper posits similarities and differences in cross-cultural workplace ageism—a scholarly sub-area which, as a whole, largely lacks theoretical frameworks. Such a perspective also allows researchers to go beyond ascertaining global attitudes toward older adults, as prior work has established (North and Fiske, 2015b; Ackerman and Chopik, 2021), and toward more nuanced understanding of how and why age-based attitudes form.

## Cross-Cultural Ageism: A Brief Overview

It is widely known that the worldwide population is aging globally. By the year 2050, an estimated two billion people will be over age 60, up from 900 million in 2015 (World Health Organization [WHO], 2018). Economically speaking, some conceptualize worldwide population graying as a primary harbinger of economic recession (Bloom et al., 2010). Others argue more optimistically that older adults remain the world’s last natural growing resource, and thus rife with novel economic opportunities (North, 2015). Regardless, the United Nations has declared the issue one of primary worldwide concern (United Nations Department of Economic and Social Affairs, 2020).

Nevertheless, these population aging trends are not uniform across nations. In East Asia, especially, population aging has experienced more recent dramatic spikes—which prior research has linked with negative attitudes toward older adults (Löckenhoff et al., 2009). Such research links also higher levels of Eastern collectivism with negative attitudes toward older adults—suggesting that in societies emphasizing the need to care for others, enlarged older populations may be seen as more burden than boon (North and Fiske, 2015b). Further complicating the matter, other recent work suggests that Eastern cultures report more positive attitudes toward older adults, at least as gauged by the Implicit Association test and an explicit, one-item preference measure of older adults to younger adults (Ackerman and Chopik, 2021). Possible explanations for differences emerging between the Löckenhoff et al. (2009) and Ackerman and Chopik (2021) findings are: significant differences in time of data collection; the disparate age range of the samples studied (the former comprised adults ages 15–89; the latter, college students); and operationalization of ageism (the latter’s focus on nine dimensions of age perception—e.g., “attractiveness,” “general knowledge”—offers more multidimensionality than the former).

In any case, the overall picture of cross-cultural ageism remains a bit murky, suggesting a need for a more nuanced approach to unpacking the extent to which age *per se* drives prejudice, versus other, age-related factors. As a starting point, the current paper focuses on China and the United States as prototypes of Eastern and Western cultures, even though it is clear that actual East and West comparisons comprise far greater complexity.

## A *GATE* to Understanding Older Workers: How Generation, Age, Tenure, and Experience Shape Workers’ Outlook

Although research on age in the workplace has garnered increased scholarly attention in recent years (Truxillo et al., 2015), some ambiguities remain. In particular, researchers have increasingly questioned the reliability of chronological age as a predictor of key work outcomes to begin with, as chronological age does not tend to predict most core work performance domains (Ng and Feldman, 2013a,b). A similar paradox afflicts the domain of prejudice and discrimination, whereby older workers are ostensibly valued for their experience (Pitt-Catsouphes et al., 2007) and yet simultaneously face particularly high levels of exclusion, in the labor market and on the job (Lahey, 2008; North and Fiske, 2016).

In order to solve this dilemma, a recent perspective advocates for disentangling between various age-based predictors: Generation, Age (lifestage), Tenure, and Experience [GATE; (North, 2019)]. Among other contributions, GATE clarifies how workplace “age”-ism emerges, as the following section discusses.

### Generation

The concept that birth cohorts pass through time and form a distinct, shared “generational consciousness” based upon formative events stems from classic sociological theory (Mannheim(ed.), 1928/1952). In the workplace, by extension, scholars posit that birth cohorts bring these values to the workplace, fostering differences (Joshi et al., 2010, 2011; Twenge, 2010). Although some empirical evidence supports the veracity of generational-bracket differences in the workplace (Twenge and Campbell, 2008), recent skepticism has clouded the perceived veracity of generational differences (Rudolph and Zacher, 2017). Nevertheless, what most scholars currently agree upon is that *perceptions* of generational differences likely hold more water, much in the same way that age stereotypes exist, regardless of the accuracy of such perceptions (Lester et al., 2012; North and Shakeri, 2019). Scholarly debates notwithstanding, popular narratives continue to emphasize the idea that workers of different birth cohorts differ in their values. In the United States, these perspectives center upon the five current, proverbial workplace generations: “Silents” (born between 1928–1945); “Boomers” (1946–1964); “Generation X” (1965–1980); “Gen-Y/Millennials” (1981–1996); and “Generation Z” (post-1997; Dimock, 2019). Whether these brackets pervade countries and cultures around the world is debatable at best, given vastly different, country-specific circumstances (e.g., economic prospects; Kearney, 2018). Nevertheless, perhaps for lack of a more mainstream alternative, scholars do often adopt these brackets when attempting to compare generations across cultures (e.g., Prabhu et al., 2017).

### Age (Life Stage)

Scholars historically have utilized life stage theories to explain a host of psychological phenomena. Classic developmental work—much of which serves as the basis of lifespan development theory, guided by the assumption that development is a lifelong process that does not stop in adulthood (Baltes et al., 1999)—posits that people progress through a series of key stages, each comprising a personal crisis to be resolved. For instance, Erikson (1963) posited that younger adults reconcile their relative place in society and relationships (identity vs. intimacy); then, middle-agers ponder how to make their mark on their external environment (generativity); and finally, in older age, people come to terms with their life and relative place in the world (ego integrity). By the same token, organizational scholars suggest that work motivations shift over time, from early career challenge and advancement, to mid-career stability and maintenance, to late-career decline and disengagement (Super, 1980).

### Tenure

Tenure reflects simply the amount of time that a worker has spent with their current organization (Staw, 1980). Although highly correlated with chronological age, tenure fosters distinct work cohorts that (for example) separate the “old guard” from the “newcomers,” regardless of chronological age or generational membership. Tenure-based divisions often outpace chronological age in predicting the closeness of interpersonal ties, due to the years of shared experience that fosters unique trust and organizational culture (Lawrence and Zyphur, 2011). Classic work in organizational demography—essentially an organization’s tenure distribution of workers—argues that tenure variance is a primary driver of organizational culture, or the degree to which norms and values are shared throughout (Carroll and Harrison, 1998).

### Experience

Unlike tenure, which quantitatively reflects amount of time with an organization that shapes internal organizational values and prescribed behaviors, experience is more qualitative, reflecting a worker’s honed knowledge, skills, and abilities accumulated over time (Quińones et al., 1995; Tesluk and Jacobs, 1998; Sturman, 2003). The value of experience as a means of enhancing abilities derives from several classic perspectives that do not necessarily cover age directly, including Human Capital theory (Sturman, 2003), learning theory (Kolb and Kolb, 2009), and psychology of aging [in which crystallized intelligence offsets declines in fluid intelligence; North and Fiske (2012); Salthouse (2012)].

## Unpacking Cross-Cultural Ageism as *GATE*-ism

Although cross-cultural ageism research in general warrants greater attention, GATE can help provide a framework to disentangle highly intertwined, age-related factors that each drive prejudice.

In outlining the utility of a GATE perspective, for each GATE dimension, I provide an overview of (a) how existing research suggests that the focal GATE dimension drives prejudice toward older adults; (b) outline how Easterners and Westerners potentially differ in their conceptions of the focal GATE dimension, and (c) speculate how Easterners might differ from Westerners in their nature (or level) of prejudice based on the focal GATE dimension.

### “Generationism” Across Cultures

#### Generation-Based Prejudice Findings (General)

As noted, generational attributions have proliferated in recent years, particularly as the number of co-existing generations has spiked. Scholars often base their research perspectives on proverbial generational brackets (Boomers, Millennials, and so on), using these distinctions to compare work outcomes between them. For instance, evidence suggests that older *generations* are viewed more positively than comparable older *age groups* (Weiss and Zhang, 2020). This result dovetails with prior work showing that it is generally adaptive for older adults to identify with their generational membership, rather than their life stage (Weiss and Lang, 2012).

#### Eastern Versus Western Generation Conceptions

Nevertheless, some argue that applying United States-based generational brackets to Eastern cultures fails to capture the nuances associated with formative generational events. This is especially the case in China, where, for instance, the One-Child Policy (whose enforcement began in 1980) uniquely shaped generational outlooks in a way that did not affect the United States (Scharping, 2013).

Instead, such critics argue that Chinese brackets of late should comprise 10-year increments more than 20-year ones (Dychtwald, 2021). These increments are based upon differing family structures (influenced by the aforementioned one-child policy), economic prospects, and technological changes (Pan, 2017; Dychtwald, 2021; Lien et al., 2021). An early perspective on Chinese generations argues that the four oldest comprise Republicans (born 1930–1950), Consolidation (1951–1960), Cultural Revolution (1961–1970), and Social Reform (1971–1980) (Egri and Ralston, 2004). Given that these four brackets are based on a 1995 survey, more modern perspectives argue that generations born in the 1980s, and 1990s, respectively comprise distinct, additional generations of the “Post-80s” and “Post-90s” (Tang et al., 2017; Gao et al., 2021). In total, these prevailing perspectives comprise six total working Chinese generations, compared to five American ones (see Table 1). Notably, cross-generational differences in work values appear to be significantly larger within the United States workplace than the Chinese one (Yi et al., 2015).

**TABLE 1:** Unpacking GATE (Generation, Age, Tenure, and Experience) Differences in China vs. the United States.

GATE dimension	Chinese conceptions	United States conceptions	Possible prejudice differences
Generation (birth cohort)	Republican (born 1928–1946)	Silent (born 1930–1950)	• Focus on attitudes toward American “Boomers” may not apply to the East • Proverbial, 20-year Western-based brackets may be better analogized to 10-year brackets in China • Prevailing (Western) views argue that generations matter in fostering workplace perceptions more than realities; remains to be seen if same holds true in the East
	Consolidation (1951–1960)		
	Cultural Revolution (1961–1970)	Boomer (1964–1964)	
	Social Reform / Oilinghou (1971–1979)	Gen-X (1965–1980)	
	Post-80s / Balinghou / Reform (1980–1989)	Millennial (1981–1996)	
	Post-90s / Jiulinghou / Post-Reform (1990–1999)	Gen-Z (1997–)	

Age (life stage)	Focus on developmental milestones that are collectivistically focused	Focus on developmental milestones at certain ages that are more individually-focused	• Perceived “failure” to achieve individual (Western-driven) developmental milestones might reflect success in Eastern, collectivistic contexts • Western research indicates that workplace “age”-ism driven by life-stage beliefs (decline of abilities) differentially targets young-old vs. old-old… • …but it remains to be seen if the same holds true in the East, where workers traditionally do not work past 60

Tenure (work cohort)	Filial piety expectations demand loyalty (versus the West) to current organization	Expectations of loyalty but also understanding that younger workers tend to “job-hop”	• Chinese (versus Western) filial piety expectations more fundamentally ingrained in organizational processes… • …but stricter mandatory retirement laws/policies inhibit senior workers to remain for a long period of time • Unclear whether junior workers punished more harshly in the East for job-hopping, given filial piety expectations

Experience (skill-shaping life and work events)	Filial piety expectations mixed with stricter regulations constricting the ability to work into later life	Employer value placed on experience but documented evidence of increased age discrimination	• Stricter mandatory retirement laws/policies in the East make it unclear whether/how experience is valued in modern Chinese organizations

#### Western Versus Eastern Generationism

Western perspectives on age-based prejudice stemming from generational membership (“generationism”) primarily emphasize perceptions of “Boomers” (or, “Millennials,” when unpacking age-based prejudice targeting the young; Francioli and North, 2021). Most prominent in these approaches has been exploring prejudicial attitudes toward the American Boomer generation. Emergent research shows largely that evaluators denigrate “Boomers” more than “older workers” (North, 2019). For instance, Boomers are perceived as competitive and indulgent, whereas older workers are seen as dependable (Posthuma and Campion, 2009; Perry et al., 2013; Costanza and Finkelstein, 2015). In the same vein, Boomer workers are more likely to be fired, whereas older workers are more likely to be hired and defended from derogatory jokes (Cox et al., 2018).

In China, the “Consolidation” generation might seem to reflect the closest parallel to American Boomers in birth cohort and related come-of-age circumstances. Like Boomers, this generation resulted from a Baby Boom, and enjoyed greater economic security than prior generations (Egri and Ralston, 2004). Nevertheless, far more differences emerge between American Boomers and Chinese Consolidationists For instance, depictions of United States Boomers revolve around a strong work ethic, competitiveness, individualism, and social liberalism, whereas Chinese Consolidationists are considered more collectivistic, idealistic and socially conservative (Markus and Kitayama, 1991; Egri and Ralston, 2004; Tang, 2019). Thus, equating the two subsets of today’s older workers appears problematic, at best. A similar level of inconclusiveness has emerged with respect to studies on Gen-Z, the youngest currently working generation, in which Eastern-born students show more individualistic mentalities than prior generations (Qian and Liu, 2021).

Nevertheless, whether these “realities” map onto *perceptions* of these generations to foster age prejudice remains an empirical question. Unlike the abovementioned studies linking negative perceptions of the American Boomer generation, by contrast, it is less clear that Chinese workplace generations face particular stereotypes stemming from their perceived generational membership *per se*. Much of the scholarly work on ageism in China tends to focus on the perception of modern older adults as burdensome, resource-depleting, and non-contributing (Bai et al., 2016). Nevertheless, the extent to which this perception is driven by perceptions of the Chinese Consolidation or Cultural Revolution *generational* brackets *per se* (i.e., a cohort effect), versus later-life-stage impairments that render them seemingly non-contributive (i.e., life-stage effect) remains unclear. Thus, the extent to which generationism is a uniquely Western phenomenon—not to mention what generational brackets make the most sense in studying this cross-cultural research question—is an area ripe for future research (see Table 1).

### “Lifestage-ism” Across Cultures

#### Lifestage (Age)-Based Prejudice Findings (General)

From an age prejudice perspective, much of what scholars have, to date, dubbed “ageism” in many ways reflects a conception of “life-stage-ism.” Classic ageism explanations largely fall into this category (North and Fiske, 2012): Terror management theory explains ageism *via* mortality salience—the fear of death associated with older age (Greenberg et al., 2002); sociological perspectives cite the advent of the printing press as reducing the record-keeping, storytelling role of later-life-stage adults (Nelson, 2005); evolutionary frameworks point to anxieties associated with later-life illness (Kurzban and Leary, 2001); and resource competition-based theories posit that younger adults in later life stages are seen as less in need of desirable assets (North and Fiske, 2013a). Overall, theorists tend to posit that older adults (and, by extension, older workers) face “age” prejudice (and related discrimination) primarily due to later-life-stage concerns: perceived lack of ability, health, and deservingness. Nevertheless, a caveat to some of these theories is the recent finding that modernization tends to predict older adults’ subjective social status (Vauclair et al., 2015).

#### Eastern Versus Western Lifestage (Age) Conceptions

As noted, lifestage development theories are predicated on the basic concept that all human beings progress through a series of steps from childhood through late adulthood. Such theories tend to span Eastern and Western cultures; for instance, the Western-defined tenet that developmental progression entails resolving specific crises (Kohlberg, 1976; Erikson, 1993) is a concept often applied to studying development among Easterners, as well (Zhang L., 2013; Li and Xu, 2018). Nevertheless, some evidence suggests that cross-cultural differences exist among children, both in the development of Eriksonian stages (Wang and Viney, 1996) and Kohlberg moral development stages (Tsui and Windsor, 2001). At the adult level too, similar differences emerge, largely due to the proverbial Western-individualism-versus-Eastern-collectivism distinction that presumably affects development in disparate ways (e.g., achieving autonomy signals aptly fulfilling a key Western developmental stage; whereas in the East, the same behavior might indicate immaturity and a failure to harmonize with family; Greenfield et al., 2003). Thus, some degree of debate concerns whether Chinese developmental psychology should apply Western theories, versus employing uniquely Chinese perspectives to understanding psychological development (Tardif and Miao, 2000).

#### Western Versus Eastern Lifestage (Age)-ism

However, whether lifestage-driven “age” *prejudice* persists across cultures is a more open question—one that is more unresolved than it might seem at first glance. In the Western view, for instance, a critical distinction entails the usually still-working, still-active “young-old” (roughly 55–75 years old) versus the less active, usually retired “old-old” (Neugarten, 1974; North and Fiske, 2013b). From this standpoint, these different life stages likely present different forms of “age”-ism—namely, expectations to actively step aside, reflecting *Succession*,” targeting the young-old, versus passive *Consumption* expectations to minimize shared resource use, targeting the old-old (North and Fiske, 2013a,b; Martin and North, 2021). However, in the Eastern context, such differences are unlikely to emerge, at least not in the same manner, due to the older workers not tending to work as long across the lifespan. For instance, China’s average retirement age is 54, and current mandatory retirement policies top out at age 60—both well before “old-old’ life stage is reached (The Economist, 2021).

Recent research further supports cross-cultural differences in lifestage-driven perceptions, centering on East-West differences in attitudes toward older adults. On one hand, lay beliefs argue that Eastern cultures tend to endorse greater collectivistic expectations to care for one’s elders (Nelson, 2009). Supporting this, as cited earlier in this paper, at least one recent study shows that Eastern (versus Western) participants report more positive implicit associations with older adults, and explicitly rate them more positively on a one-item preference measure (Ackerman and Chopik, 2021). On the other hand, a recent meta-analysis contradicts many of these assumptions, finding instead that Eastern cultures harbor more *negative* views of later-life-stage adults, and that these perceptions are predicted by heightened collectivism and recent spikes in population aging (North and Fiske, 2015b). Further complicating cross-cultural examinations, the warm-but-incompetent stereotype of later-life-stage adults often cited in Stereotype Content Model research (Fiske et al., 2002) also appears to be pan-cultural (Cuddy et al., 2009). Thus, greater cross-cultural research attention—especially that which isolates life stage as a driving mechanism in fostering workplace ageism—will help clarify these inconsistencies.

### “Tenure-ism” Across Cultures

#### Tenure-Based Prejudice Findings (General)

Despite lying largely outside of what scholars typically refer to as “ageism,” prejudices arising from organizational tenure are surprisingly potent and prevalent. Tenure-based differences (e.g., the “old guard” who entered the organization a similarly long time ago, versus newcomers who entered more recently) are among the strongest factors driving workplace conflict, social exclusion, communication breakdowns, turnover, and information withholding (McCain et al., 1983; Zenger and Lawrence, 1989; Lawrence and Zyphur, 2011; Gilson et al., 2013). The strong impact of tenure-based organizational demography (as described earlier in this paper) gives rise to organizational *faultlines*—perceptions of strong subgroup differences (Lau and Murnighan, 1998). Such faultlines can be particularly risky in (unhealthy) task conflict spilling over into (unhealthy) relationship conflict, undermining group productivity, especially when tenure faultlines interact with age faultlines (Choi and Sy, 2010). Nevertheless, various organizational scholars have argued that tenure-based relationships are stronger than those based on gender, ethnicity, or any other social category, including chronological age (McCain et al., 1983; Pfeffer, 1983, 1985; Lawrence and Zyphur, 2011). For these reasons, one could argue that within the work context, prejudices arising from tenure differences drive a good portion of what researchers traditionally refer to as “ageism” (North, 2019).

#### Eastern Versus Western Tenure Conceptions

Expectations concerning organizational tenure tend to differ between Eastern and Western cultures for two key reasons. The first factor entails differential levels of emphasis on the Confucian ideal of filial piety—that is, loyalty to one’s family (Bedford and Yeh, 2019) and by extension, one’s employer or work supervisor (Hwang, 1999; Chen et al., 2002; Zhang C., 2013). In the Eastern context, studies often implicate filial piety expectations with key organizational outcomes, including organizational commitment, work satisfaction, work motivation, and supervisor-subordinate exchange (Jen et al., 2012; Lee et al., 2018; Liu et al., 2020; Li et al., 2021). Such findings dovetail with consensus beliefs that filial piety expectations, at least from a moral obligation standpoint, more strongly occur within Eastern versus Western societies (Ni, 2020). This all implies—as research supports—the idea that filial piety expectations likely underlie organizational processes more in the former than the latter.

A second factor underlying likely East-West differences in tenure expectations is variance in retirement policies. As noted, Eastern societies tend to uphold stricter standards of mandatory retirement, at least compared to the United States. With China’s current policies dictating the retirement of employees after age 60 (The Economist, 2021), the ability of the “old guard” to stick around is more limited than in the West, in general (see Table 1).

#### Western Versus Eastern Tenure-ism

Sparse research directly compares Eastern and Western cultures in tenure-driven prejudices. Nevertheless, it is logical to believe that distinct levels of “tenure”-ism might emerge, given the abovementioned East-versus-West differences in tenure expectations (The Economist, 2021). Eastern filial piety emphasis, combined with shorter tenure opportunities and increased expectations the old guard eventually steps aside at a predictable point, might reduce “tenure-ism” in the East. By contrast, in the West, it is often the ambiguity surrounding precisely when the old guard will make way for younger generations that drives generational tensions (North and Fiske, 2013a). However, future research is needed to establish potential cross-cultural differences in prejudice thereof.

Complicating the picture further, there is reason to believe that the opposite pattern may be true when it comes to attitudes toward younger workers—that is, Easterners may be more harsh on junior workers who violate tenure expectations. As one concrete example, the current younger United States generation often faces resentment for perceptions of increased “job hopping” behaviors (Francioli and North, 2021). It is unclear whether the same would be true in the Eastern context. Nevertheless, given Eastern loyalty expectations, such behavior might be seen as extra deviant, and thus resulting in greater backlash. By comparison, job hopping might be seen as more sanctioned in the West, where individualism is valued, and the practice of exploring options is believed to lead to an increase in personal fulfillment (Meister, 2012; Table 1). Although scholars have begun to posit that the same expectations may not apply to Eastern cultures (Raina and Chauhan, 2016), more focused research attention must establish this conclusively.

### “Experience-ism” Across Cultures

#### Experience-Based Prejudice Findings (General)

Scholars and practitioners alike often cite experience as a key benefit of older workers (Pitt-Catsouphes et al., 2007; Paullin, 2014). In line with this, organizations tend to equate seniority with hierarchical position (Fischer, 2008). At the same time, experience can offer a downside in workplace treatment, fostering perceptions of excess cost or inability to train in new skills (Brooke and Taylor, 2005; Porcellato et al., 2010). From this latter standpoint, the old adage, “you can’t teach an old dog new tricks” is often what underlies workplace “age”-ism—implying that with age comes an increasingly crystallized, immutable skill set. At the group level, too—and resembling the aforementioned prejudices that can emerge *via* different levels of tenure—similar results imply that experience-based differences also foster division. Although in some cases variance in experience can enhance an organization’s strategic flexibility (Heavey and Simsek, 2017) and performance (*via* allocation of skill specialization; Bunderson, 2003), differences in experience levels can give rise to communication breakdowns, conflict, and turnover in the workplace (Zenger and Lawrence, 1989; Backes-Gellner and Veen, 2013).

#### Eastern Versus Western Experience Conceptions

Much like the case of organizational tenure described earlier in this paper, stricter mandatory retirement laws in the East likely foster differences in the extent to which workers’ experience is valued. Indeed, in spite of society-level filial piety expectations to respect and include elders, Eastern *organizations*, by contrast, tend to marginalize older workers, in spite of their experience (Chiu and Ngan, 1999). This suggests that within organizations, older workers’ experiences may not be particularly valued in comparison to the West. In fact, some argue that the situation may be worse as Eastern societies increasingly modernize (Cheung and Kwan, 2009).

Some hold out hope that cultural emphasis on life experiences, combined with a knowledge economy and a declining workforce, might increase the premium placed on older workers in the East (Chan and Liang, 2013). Nevertheless, proverbial norms of retiring by age 60, regardless of statutory retirement age (which does vary across different countries), threaten this belief, as well (Egdell et al., 2019). Much as in the West, although older workers’ experience is recognized in the East, so too are negative perceptions of that same experience inhibiting training ability, willingness to relocate, and motivation for innovating (Lu et al., 2011; Table 1).

#### Western Versus Eastern Experience-ism

As with the other research trajectories covered here (generation, lifestage, and tenure), more research is needed to directly compare Eastern and Western workplaces on how much they might discriminate on the basis of experience *per se*. Nevertheless, in light of the aforementioned findings, it would be shortsighted to assume that Eastern workplaces value experience more, as whatever social norms to respect seniority dictate, they are likely outweighed by practical forces (e.g., rigid age-based retirement expectations). Compared to prior eras, Eastern economies are operating within particularly steep rates of population aging (Eberstadt, 2009; Bloomberg Data, 2012) and industrial change (McMichael, 2017). Thus, older worker experience may not be as valued as it once was (Cheung and Kwan, 2009; Table 1). Future research should establish more conclusively the way in which experience is valued in Eastern versus Western organizational contexts.

## Conclusion: A *GATE* to Unpacking Cross-Cultural Workplace Ageism

The global workplace is growing older and more multi-generational (Kulik et al., 2014; North and Fiske, 2015a), and these trends encompass similarly industrialized Eastern and Western societies. Therefore, how older workers will be valued, and how generations will work together, is set to become a defining issue of cross-cultural management. However, age-based prejudices threaten this growing need, as does theoretical ambiguity concerning how researchers can most precisely study and understand age differences. Toward these aims, a GATE (generation, age, tenure, and experience) framework helps disentangle age-related factors into more nuanced components, setting up promising research trajectories for better understanding cross-cultural workplace ageism. Utilizing these pathways, scholars can eventually understand how workplace age prejudice can best be mitigated regardless of cultural context (Marcus and Fritzsche, 2016), toward less ageist, more generationally inclusive workplaces around the world.

## Author Contributions

MSN contributed to all aspects of the manuscript.

## Conflict of Interest

The author declares that the research was conducted in the absence of any commercial or financial relationships that could be construed as a potential conflict of interest.

## Publisher’s Note

All claims expressed in this article are solely those of the authors and do not necessarily represent those of their affiliated organizations, or those of the publisher, the editors and the reviewers. Any product that may be evaluated in this article, or claim that may be made by its manufacturer, is not guaranteed or endorsed by the publisher.
